# Evaluation of an experimental model of senescence SAMR1/SAMP8 for microRNA studies in acute myocardial infarction: role of sex and aging

**DOI:** 10.1007/s13105-026-01194-y

**Published:** 2026-05-23

**Authors:** A. B. Paes, B. Descals-Beltrán, C. Rosales-Ariza, A. Díaz, D. Pérez-Cremades, A. P. Dantas, C. Hermenegildo, S. Novella

**Affiliations:** 1 Department of Physiology, University of Valencia, INCLIVA Biomedical Research Institute, Valencia, Spain; 2 Central Biomedical Research Unit (UCIM), University of Valencia, Valencia, Spain; 3 Department of Biomedical Sciences, School of Medicine and Health Sciences, Institut D’Investigacions Biomediques August Pi I Sunyer, University of Barcelona, Hospital Clinic Cardiovascular Institute, Barcelona, Spain

**Keywords:** Aging, Myocardial infarction, miRNA biomarkers, Sex differences, SAMP8 mouse model

## Abstract

Age and sex are critical yet often overlooked variables in preclinical models of acute myocardial infarction (AMI), potentially limiting the translational relevance of molecular findings. In this study, we used the Senescence-Accelerated Mouse Prone 8 (SAMP8) strain, a model of age-related cardiovascular dysfunction, alongside age-matched SAMR1 controls, to investigate early expression patterns of four canonical circulating miRNA biomarkers of AMI: miR-1-3p, miR-133a-3p, miR-208a-3p, and miR-499-5p. Male and female mice underwent left anterior descending (LAD) coronary artery ligation, with serum and cardiac tissue collected at 1, 4, and 24 h post-injury. Results reveal a robust early induction of all four miRNAs in serum, with 4 h identified as the optimal profiling time point. Notably, miR-1-3p was significantly elevated in both male and female SAMP8 mice post-AMI, but not in SAMR1. miR-133a-3p increased only in aged females, while male SAMP8 and SAMR1 mice showed divergent responses. miR-208a-3p and miR-499-5p rose consistently across all groups. In cardiac tissue, SAMR1 mice exhibited downregulation of all four miRNAs, whereas only miR-1-3p was reduced in SAMP8 males. Baseline expression in sham-operated hearts revealed age-related differences, particularly in males, with lower levels of miR-133a-3p, miR-208a-3p, and miR-499-5p in SAMP8 versus SAMR1. These findings highlight pronounced age- and sex-dependent miRNA expression dynamics in both serum and cardiac tissue during the acute phase of AMI. These results validate the SAMP8/SAMR1 model as a valuable tool for dissecting molecular responses to myocardial injury and underscore the need to integrate age and sex into preclinical AMI research.

**Supplementary Information:**

The online version contains supplementary material available at 10.1007/s13105-026-01194-y.

## Introduction

Acute myocardial infarction (AMI) represents the most severe manifestation of coronary artery disease and remains one of the leading causes of mortality worldwide. Both sex and aging influence the incidence of AMI, with distinct patterns observed in clinical outcomes and presentations. Women have a lower overall incidence of AMI than men across all age groups [1], but exhibit a higher risk of adverse outcomes post-AMI, especially in cases of non-obstructive coronary artery disease (MINOCA) [1, 2]. Despite the complex interplay between sex, age and cardiovascular health, many experimental animal models of AMI fail to incorporate these variables, potentially skewing research outcomes, particularly those related to transcriptomic changes.

microRNAs (miRNAs) are small non-coding RNAs, highly conserved molecules that regulate gene expression post-transcriptionally [3, 4]. Circulating miRNAs have garnered significant attention as potential biomarkers and therapeutic targets in cardiovascular diseases [5]. Specific miRNAs, such as miR-1, miR-133a, miR-208a, and miR-499, are notably elevated in serum and plasma following myocardial infarction [6–9]. Among other functions, these miRNAs have been implicated in cardiogenesis, supporting cardiomyocytes differentiation, and cardiac conductance and contractility [10, 11], but also in the pathological reprogramming of fibroblasts into cardiomyocytes [12] and the development of cardiac hypertrophy [13], thereby altering heart function.

Despite advancements in pharmacological strategies and a deeper understanding of AMI over recent decades [14], there remains a critical need to reduce cardiac hypertrophy and fibrosis post-AMI, enhance vascularization, and prevent cardiac dysfunction. Gaining further insights into the underlying mechanisms, particularly transcriptomic changes, is essential for developing new personalized and targeted therapies. In this context, the exploration of new experimental models is crucial. Animal models are indispensable for studying the molecular basis of these processes, providing a controlled environment to investigate the temporal dynamics of transcriptomic and epigenetic modifications following AMI. These models also facilitate the exploration of novel therapeutic approaches and the evaluation of their efficacy. However, observations derived from younger animal subjects may not adequately reflect the intricate complexities of sex differences that are evident in older human patients. Thus, utilizing middle-aged and older animal models may provide a more accurate representation of human CVD.

Senescence-accelerated mouse (SAM) is a murine model commonly utilized to study age-associated diseases, including cardiovascular aging [15, 16]. Developed through phenotypic selection from breeding pairs of AKR/J mice [17], several strains of SAM mice exhibit phenotypic alterations associated with accelerated aging [18]. Notably, the SAM Prone (SAMP8) and SAM Resistant (SAMR1) strains show an aging-associated decline in endothelium-dependent relaxation and increased vasoconstrictor responses [16, 19–21]. Cardiovascular dysfunction manifests earlier in SAMP8 mice, establishing the SAMR1/SAMP8 model as suitable for studying cardiovascular aging within a practical and standardized timeframe [15, 16, 21–23]. Few studies have use SAM model to study the role of miRNA, and most of them are focused on neuronal loss and progression of age-related cognition decline [24–26].

Therefore, the aim of this methodological study was to evaluate the suitability of SAMR1/SAMP8 senescence model for the study of AMI-associated miRNA expression, and to establish an appropriate time point following infarct induction for transcriptomic analyses, as a pathophysiological model to study the effects of aging and biological sex on miRNA regulation after experimental AMI. Accordingly, this work was designed to provide a controlled experimental framework that allows the investigation of cardiac-specific miRNA regulation while minimizing the influence of confounding comorbidities commonly present in clinical settings. Based on this rationale, we focused on miRNAs previously associated with AMI, including miR-1-3p, miR-133a-3p, miR-208a-3p, and miR-499-5p.

## Materials and methods

### Experimental animals

6-month-old male and female SAMR1 and SAMP8 (*n* = 72) mice were obtained from the breeding stock at Central Medicine Research Unit of the University of Valencia and housed in a controlled environment according to institutional guidelines (22 °C, 12 h light/dark cycle, 60% humidity, and standard mice chow and water ad libitum). All animal protocols were approved by the Institutional Ethics Committee at the University of Valencia (Protocols number: 2016/VSC/PEA/00135; 2020/VSC/PEA/0128), conformed to the National Institutes of Health Guide for the Care and Use of Laboratory Animals, as well as the guidelines of the Directive 2010/63/EU from the European Parliament. Every effort was made to minimize the number of animals used and their suffering. Both SAMR1 and SAMP8 were randomly separated into two groups: sham-operated (sham) and AMI group (AMI). Sham-operated SAMR1 was used as control group, as they present a regular life-spam.

### Mouse acute myocardial infarction and sample collection procedures

Mice (25–35 g) were anesthetized by inhalation of 2% isoflurane (Abbott Laboratories, Chicago, IL, United States), and Metacam (0.3 mg/kg) and Buprenorphine (0.1 mg/kg) were administered via intraperitoneal before any surgical procedure. Endotracheal intubation without any incision was performed and mice were connected to a rodent ventilator (Minivent type 845, Panlab Harvard Apparatus, Barcelona, Spain) supplemented with 100% oxygen and 2% isoflurane at a flow rate of 0.2 L/min.

**Fig. 1:**
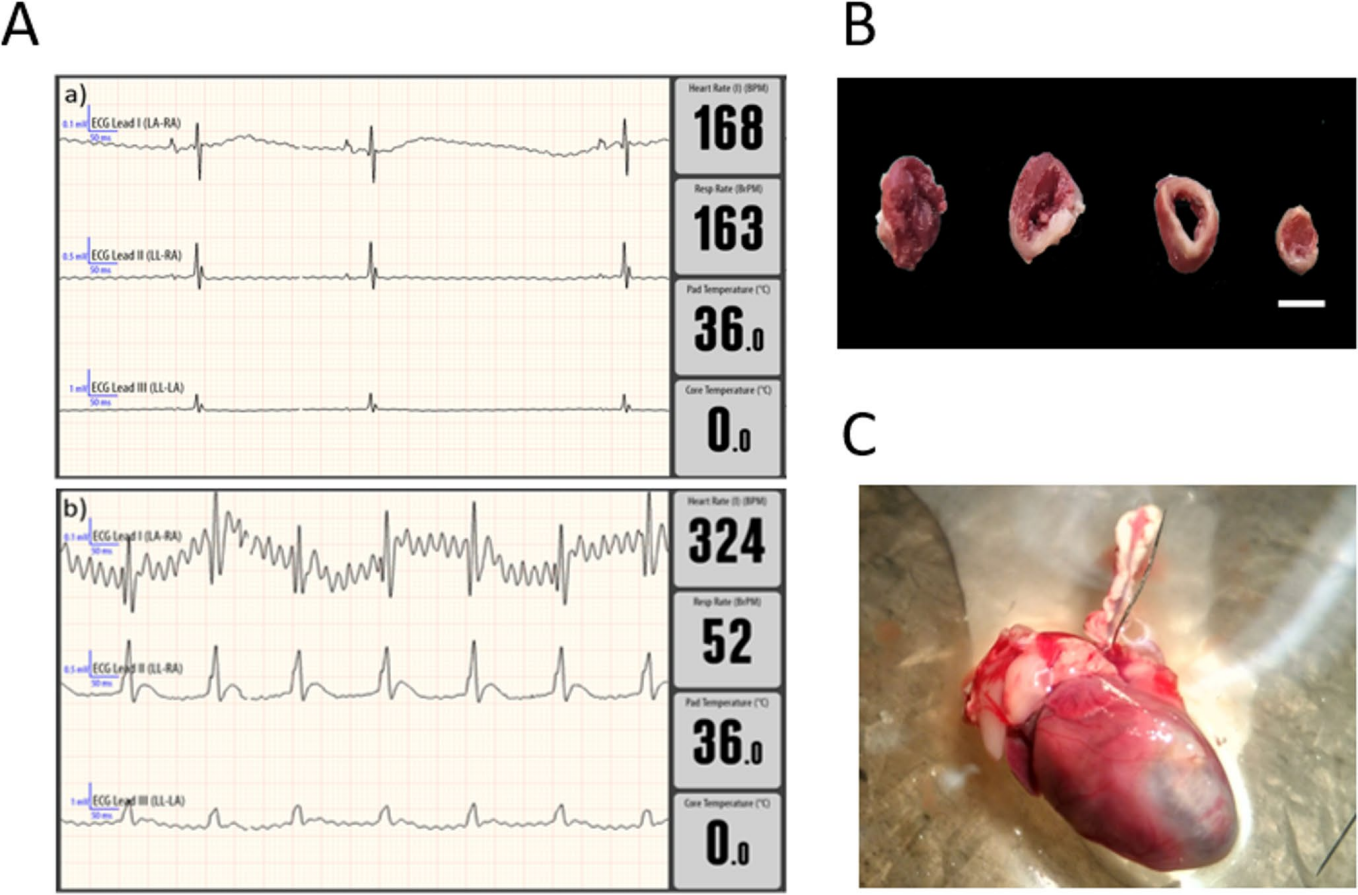
Representative electrocardiographic recording and heart staining confirming successful establishment of the AMI mouse model. (**A**) Electrocardiograms recorded after left anterior descending coronary artery (LAD) ligation (AMI group) (**b**) and sham surgery (**a**). (**B**) Representative TTC-stained heart sections from an AMI mouse 4 h after surgery. Scale bar = 5 mm. (**C**) Representative image showing left ventricular ischemia in isolated heart following AMI

Experimental animals underwent permanent left anterior descending coronary artery (LAD) ligation or a sham operation as described previously using the classical method [27, 28] achieving a survival rate higher than 80%. Briefly, a small skin cut (1.2 cm) was made over the left chest to expose the heart, and a 6 − 0 silk suture was used for permanent ligation of the LAD, approximately 1–2 mm below the base of the left auricle. Following the procedure, the thoracic cavity was closed in layers using 6 − 0 absorbable sutures for the muscle and 6 − 0 nylon for the skin, and the mice were placed in a clean recovery cage under a heat lamp to regain consciousness. AMI was visually confirmed by immediate discoloration of the left ventricle upon ligation (Fig. 1A) and ST segment elevation in in Lead II of the electrocardiogram (ECG) (Fig. 1B). Sham-operated animals underwent the same procedure without coronary artery ligation.

At 1-, 4-, or 24-hours post-surgery, mice were anesthetized with 5% isoflurane vapor. Blood samples were collected via the vena cava using a 25 G needle in an Eppendorf tube. Subsequently, animals were euthanized, and immediately thereafter, hearts were rapidly excised and processed for RNA and biochemical analyses to preserve molecular integrity. After resting for less than 1 h at room temperature, the blood samples were centrifuged to obtain serum, which was stored at -80 °C until further analysis. To evaluate the tissue viability and infarct size, histological analysis with TTC (2,3,5-Triphenyltetrazolium chloride) staining was performed. Briefly, hearts were perfused with 10 ml of PBS, excised, and immediately placed in ice cold PBS before being fixed in 10% formalin overnight at 2–8 °C and prepared 1 mm slices for TTC staining which further confirmed left ventricle ischemia (Fig. 1C). For RNA extraction, hearts were kept in RNAlater (Invitrogen, ThermoScientific) and stored at -80 °C.

### RNA extraction and real-time quantitative PCR

Total RNA was isolated from 200 µl serum using the miRNeasy Serum/Plasma Advanced Kit (Qiagen) following the manufacturer’s instructions as previously described [29]. The obtained RNA was eluted through the spin column with RNase-free water. In hearts, total RNA was extracted from small tissue fragments by mechanical homogenization and phenol-chloroform method. Briefly, tissues were mechanically homogenized in RNase/DNase-free 2 ml tubes containing a 5 mm stainless steel bead (Cat. No. 69989, Qiagen) and 1 ml of TRItidy (TRItidy G™, AppliChem GmbH). Samples were homogenized in a pre-cooled TissueLyser for two cycles of 5 min at 50 Hz. After homogenization, chloroform was added, mixed, incubated 3 min at room temperature, and centrifuged at 16,100 rpm for 15 min at 4 °C. The aqueous phase was collected, RNA was precipitated with isopropanol, washed with 75% ethanol, and resuspended in RNase-free water. RNA samples were stored at − 80 °C.

The total RNA amount and purity were measured using the NanoDrop One spectrophotometer (ThermoFisher). For miRNA detection, reverse transcription and quantitative real-time PCR (qRT-PCR) were performed using the TaqMan MicroRNA Reverse Transcription Kit (Applied Biosystems) and the TaqMan Universal PCR Master Mix (Applied Biosystems), respectively. Gene-specific primer pairs and probes (Applied Biosystems) were used for U6 snRNA (001973), miR-1-3p (002222), miR-133a-3p (002246), miR-208a-3p (000511), and miR-499-5p (001352), following the manufacturer’s instructions. The reverse transcription (RT) conditions were 30 min at 16 °C, 30 min at 42 °C, and 5 min at 85 °C. The PCR conditions were 10 min at 95 °C, followed by 45 cycles of 15 s at 95 °C and 1 min at 60 °C.

For miRNA quantification, U6 small nuclear RNA was used as an endogenous reference control, as previously described in serum [30] and in heart tissue [31]. U6 was selected based on its stable expression across experimental groups and experimental conditions. The expression levels of U6 snRNA were measured in all samples to normalize differences in RNA input, RNA quality, and reverse transcription efficiency. All qRT-PCR reactions were performed in technical triplicates, and mean Ct values were used for subsequent analyses. Reactions lacking reverse transcription products were included as negative controls. Relative miRNA expression was calculated using the 2^−ΔCt^ method, where ΔCt was determined by subtracting the Ct value of U6 snRNA from the Ct value of the miRNA of interest. When indicated, fold changes relative to the corresponding sham control group were calculated using the 2^−ΔΔCt^ method, with ΔΔCt defined as the difference between the ΔCt of the experimental and control samples.

### Analysis of miRNA targets and pathway enrichment

Bioinformatic analyses were performed to identify mRNA targets and associated biological pathways of the four studied miRNAs. Target genes were obtained using the miRPath v4.0 platform (DIANA tools; https://diana.e-ce.uth.gr/home), integrating experimentally validated miRNA–mRNA interactions from the miRTarBase 2022 database. This approach was chosen to ensure a robust assessment of biologically relevant targets supported by direct experimental evidence of miRNA binding to mRNA 3’UTR regions.

Gene Ontology (GO) enrichment analysis was conducted focusing on the Biological Process domain. Enriched GO terms were ranked according to statistical significance, and the top 10 most significant pathways were selected for visualization. A comprehensive list of all enriched GO terms and corresponding target genes associated with the top pathways is provided in the Supplementary Table 1.

### Statistical analysis

Data are expressed as mean ± SEM. In each experimental group n indicates the number of animals. For single comparisons, Student’s t-test was applied. To determine differences between multiple groups, one-way analysis of variance (ANOVA) was performed, followed by Bonferroni’s post hoc test. Results were considered significantly different at a p value < 0.05. All statistical analyses and graphical representations were performed using Prism version 8 software (GraphPad Software Inc., San Diego, CA, USA).

## Results

### Serum levels of miRNA after acute myocardial infarction

SAMR1/SAMP8 mice model of cardiovascular aging replicates most of the pathophysiological states found in human. Nevertheless, their susceptibility as an AMI model has been scarcely studied. Moreover, few studies have focused on the role of miRNA regulation after myocardial ischemic injury in aged mice. Therefore, to determine optimal time for assessing miRNA levels following experimental myocardial infarction, we first analyzed circulating expression of the miRNA biomarker candidates at three time points: 1 h, 4 h, and 24 h post-surgery.

**Fig. 2:**
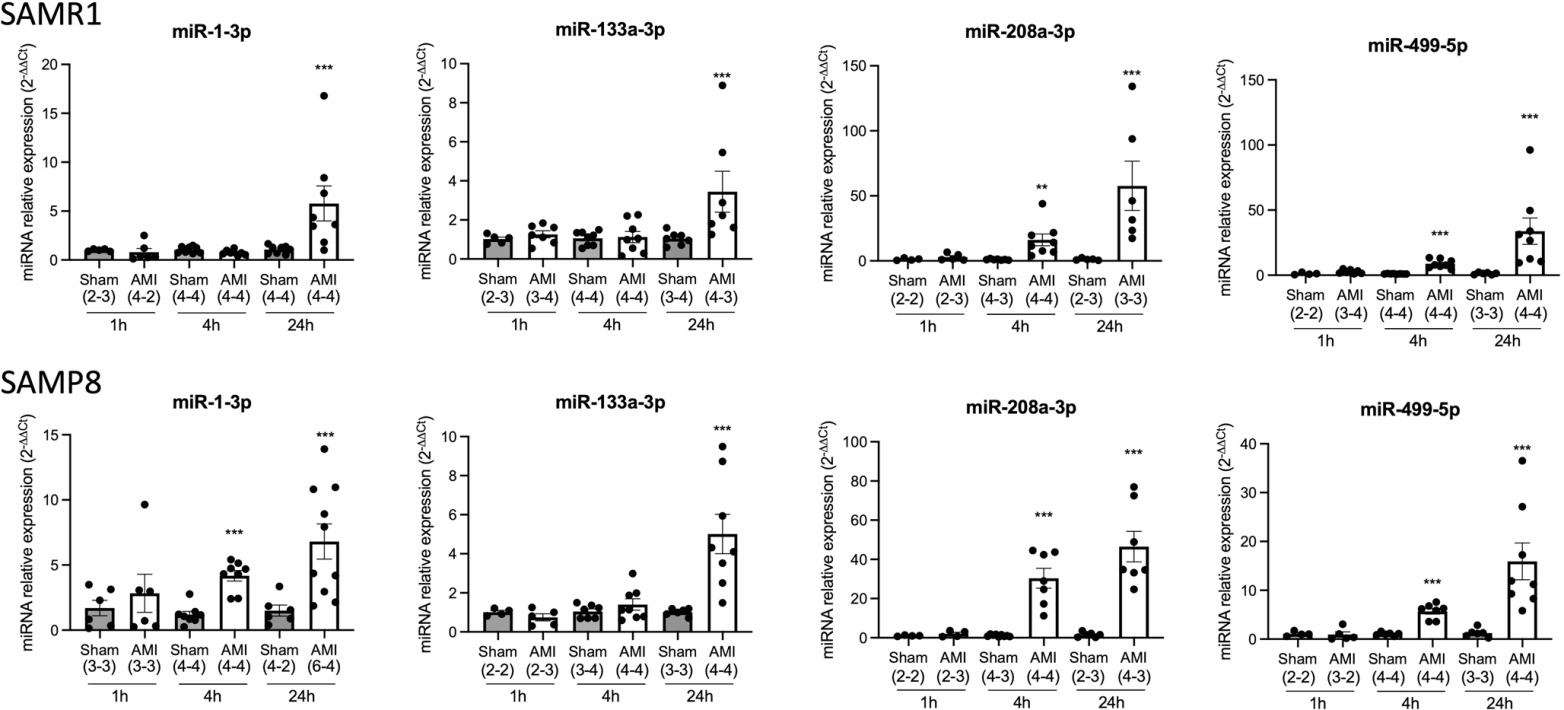
Time-course of circulating miRNA expression in serum from SAMR1 and SAMP8 mice. Serum miRNAs from both sexes were analyzed by qRT-PCR at 1, 4 and 24 h after AMI. Relative miRNA expression was calculated using the 2^-∆∆Ct^ method. Data are shown as mean ± SEM. The number of animals (n) for each group is indicated in brackets and is reported as females-males (F-M). Statistical analysis was performed using student’s t-test for comparisons between AMI vs. sham groups at each time point *p < 0.05, **p < 0.01, ***p < 0.001

Our results demonstrated that none of the miRNAs showed any significant change at 1 h time point. We observed a significant increase (*p* < 0.05) in the expression of the candidates miR-1-3p, miR-208a-3p, and miR-499-5p at 4 h post-infarction in SAMP8 mice, while only miR-208a-3p and miR-499-5p increased in SAMR1 mice. Finally, the expression of all four miRNAs, including miR-133a-3p, was significantly increased in the serum of both strains after 24 h of LAD ligation (Fig. 2), suggesting that the signalling pathways activated after AMI in this animal model are similar to those in humans. Expression changes showed in miR-133a-3p, miR-208a-3p, and miR-499-5p were similar in SAMP8 compared with SAMR1. Conversely, ischemia-induced miR-1-3p expression at 4 h was observed in SAMP8 (*p* < 0.05) but not in SAMR1 (Fig. 2), suggesting an age-related effect.

Therefore, as miRNA induction was observed as early as 4 h following ischemic injury, we selected this time point for further analysis and propose it as the appropriate time to perform transcriptomic studies in AMI in this mouse model of senescence.

**Fig. 3:**
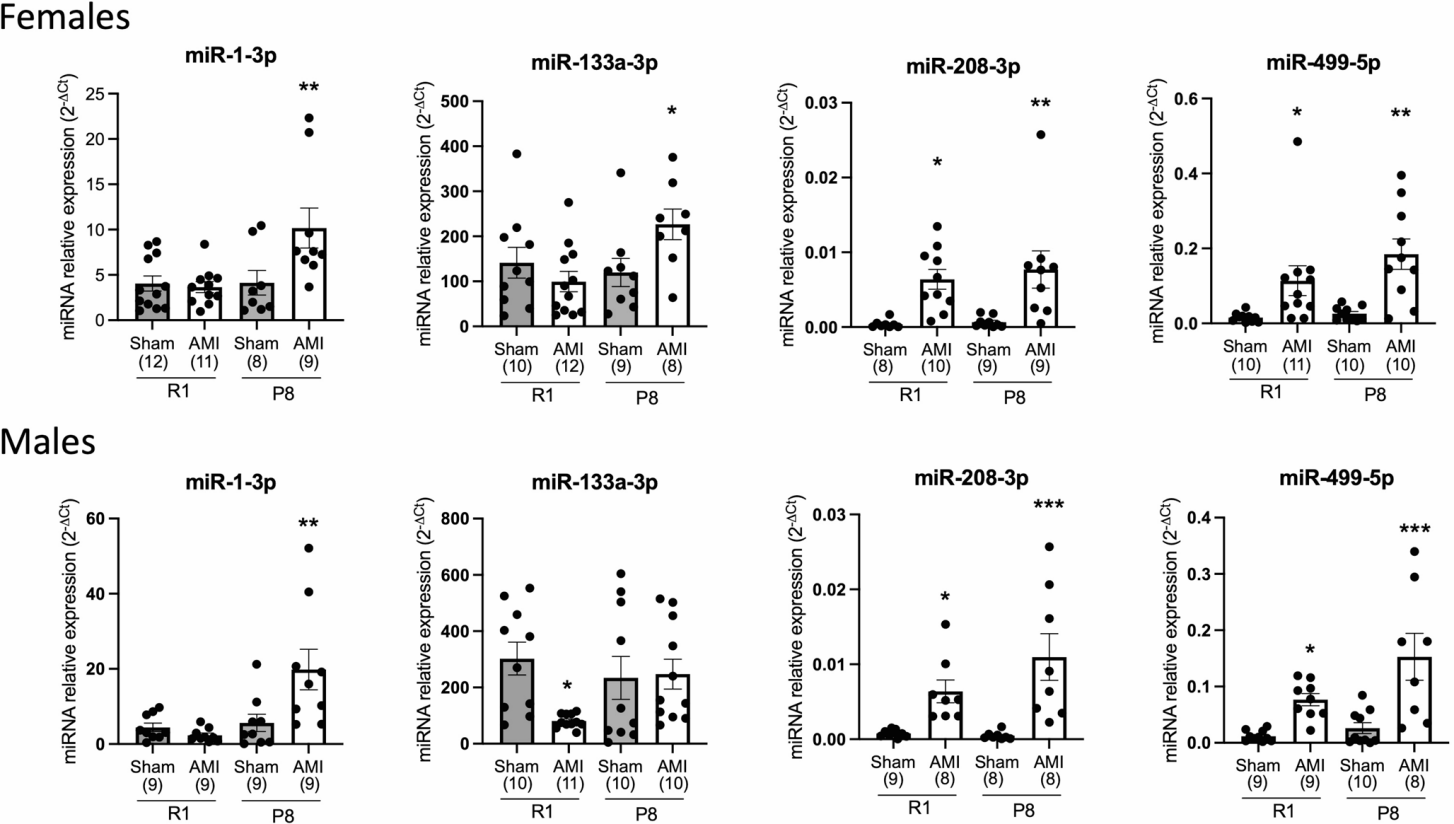
Circulating miRNA expression in serum of female and male SAMR1 and SAMP8 mice after acute myocardial infarction (AMI). Ischemic injury was induced in SAMR1 (R1) and SAMP8 (P8) mice by permanent left coronary artery ligation. Serum miRNAs were analyzed by qRT-PCR 4 h after ischemic injury. Relative miRNA expression was calculated using the 2^-∆Ct^ method. Data are shown as mean ± SEM. The exact number of animals (n) is indicated in brackets for each group. *p < 0.05, **p < 0.01, ***p < 0.001 versus sham animals of the same strain

We next determined the expression of the studied miRNAs separately in female and male mice (Fig. 3). In female mice, the expression of miR-208a-3p and miR-499-5p significantly increased four hours after LAD ligation in both SAMR1 (*p* < 0.05) and SAMP8 (*p* < 0.01) mice. Similar results that were observed for these miRNAs in male mice. Serum levels of miR-1-3p showed enhanced expression in AMI compared to sham-operated animals in SAMP8 but not in SAMR1 in both female and male mice, thus reinforcing the age-related effect on the expression of miR-1-3p after AMI. Finally, miR-133a-3p expression increased after myocardial injury in aged female mice (*p* < 0.05) but not in male mice, and was not altered in SAMR1 female mice while it decreased in male mice (*p* < 0.05), suggesting a sex difference in the expression of miR-133a-3p after AMI.

### Cardiac expression of miRNA after acute myocardial infarction

**Fig. 4:**
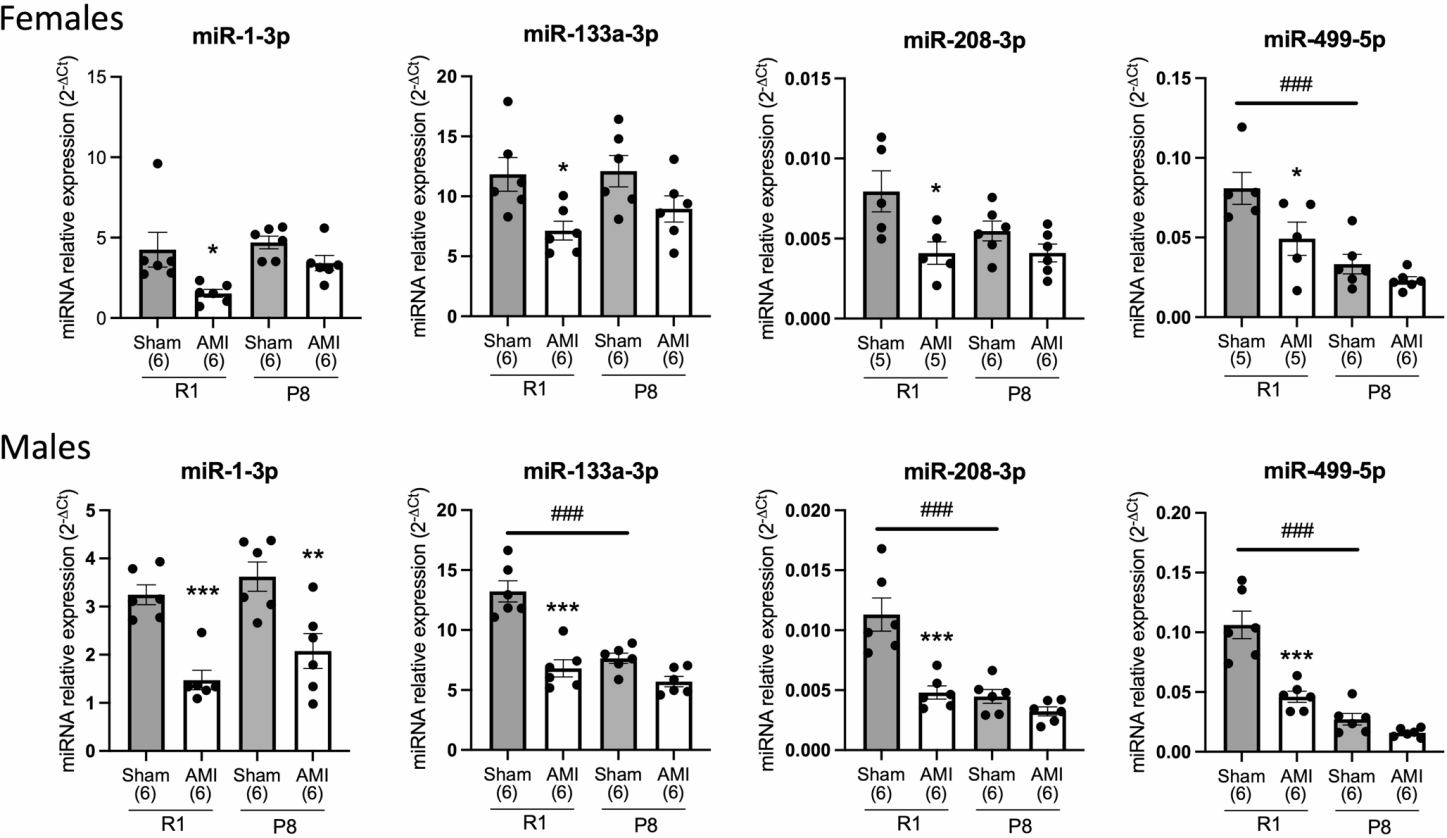
Cardiac miRNA expression in heart tissue of female and male SAMR1 and SAMP8 mice after acute myocardial infarction (AMI). Expression levels of miR-1-3p, miR-133a-3p, miR-208a-3p and miR-499-5p were analyzed in hearts from SAMR1 (R1) and SAMP8 (P8) mice 4 h after AMI. Relative miRNA expression was calculated using the 2^-∆Ct^ method. Data are shown as mean ± SEM. The exact number of animals (n) is indicated in brackets for each group. *p < 0.05, **p < 0.01, ***p < 0.001 versus sham animals of the same strain, ^#^*p* < 0.05, ^##^*p* < 0.01, ^###^*p* < 0.001 versus SAMR1 Sham

Heart-specific miRNAs are supposed to be released from the heart into the circulation during myocardial infarction. To assess whether the serum miRNAs reflect tissue expression, we performed miRNA analysis of ischemic myocardial tissue that was collected 4 h after LAD ligation (Fig. 4). In contrast to the changes observed in serum, the four miRNAs were significantly down-regulated in the SAMR1 model of AMI compared to the sham-operated mice. In SAMP8 mice, only the expression of miR-1-3p was significantly decreased (*p* < 0.01) after myocardium injury in male but not in female mice. Finally, we compared miRNA levels in sham-operated groups of SAMR1 and SAMP8 mice. In male mice, expression of miR-133a-3p, miR-208a-3p and miR-499-5p significantly decreased (*p* < 0.001) in SAMP8 compared to SAMR1 groups, suggesting an age-associated down-regulation of these miRNAs in non-ischemic hearts. However, in female mice, only the tissue expression of miR-499-5p showed a significant decreased (*p* < 0.001) in the sham-operated SAMP8 compared to SAMR1.

### miRNA targets and associated biological pathways of differentially expressed miRNAs

**Fig. 5:**
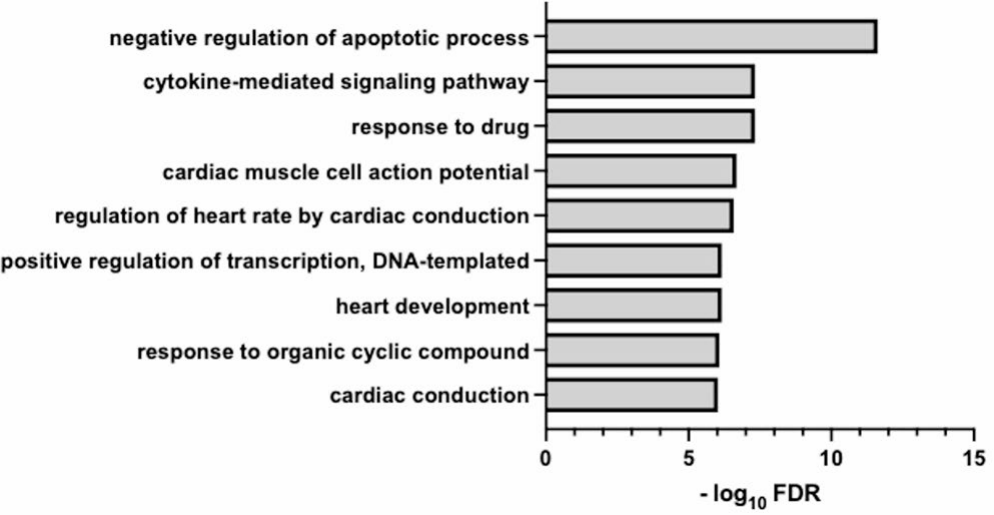
Gene ontology (GO) pathway enrichment analysis of experimentally validated targets of the miRNAs analyzed in this study. GO enrichment analysis was performed using miRPath v4.0 (DIANA Tools) based on experimentally supported miRNA–mRNA interactions revised from miRTarBase 2022, focusing on the *Biological Process* domain. The bar plot displays the top 10 significantly enriched GO terms, ranked according to the −log10 false discovery rate (FDR)

Pathway analysis based on experimentally supported targets of the miRNAs studied in this work revealed a significant enrichment of biological processes related to myocardial injury and repair. Among the top GO pathways terms within the Biological Process domain (Fig. 5), pathways associated with negative regulation of apoptotic process, cytokine-mediated signaling, and positive regulation of cell population proliferation were identified, suggesting a role for these miRNAs in the early regulation of cell survival, inflammation, and compensatory responses following ischemic injury. In addition, several cardiovascular-related processes were significantly enriched, including heart development, cardiac conduction, and membrane depolarization during cardiac muscle cell action potential. Other relevant biological processes, such as positive regulation of fibroblast proliferation, regulation of cardiac muscle hypertrophy, and wound healing, were also among the top enriched pathways (Fig. 5, Supplementary Table 1).

## Discussion

In the present study, we demonstrate the suitability of SAMP8 and SAMR1 mice as a model for myocardial infarction. We measured some well-known AMI miRNA biomarkers, and observed significant increase in circulating miRNA expression, and a reduction in heart tissue miRNA expression. In addition, the data collected using this murine model revealed interesting age- and sex-related variations in the acute phase following ischemic injury that could be relevant when study this disease.

Significant advances in the treatment of the cardiovascular diseases have emerged over the last few decades, mainly due to the accumulation of knowledge about the molecular mechanisms following myocardial infarction. This insight in the pathophysiology of the disease has mostly been gained through the use of animal models, which is a necessary step in understanding therapeutic approaches and developing drugs [32]. Occlusion of the coronary artery is probably the most well-established model for myocardial infarction. First introduced in 1954 [33], it can be easily reproduced by a surgical ligation of the LAD. With a few modifications and a skilled surgeon, the entire procedure can be complete in under 5 min per animal with a low mortality and fast recovery. The most important downfall of this intervention is the requirement for a qualified individual with extensive experience and proper training in this technique [34]. In this study, our procedure achieved a surgery survival rate of over 80%, and successful of left ventricular ischemia was evaluated by immediate discoloration of the left ventricle and elevation of the ST segment in the ECG. However, most experimental models of AMI reported to date have focused on single post-infarction time points and predominantly used young male animals, without systematically addressing the influence of aging or sex on early molecular responses.

Beyond the surgical induction of myocardial infarction, the choice of an appropriate aging model is critical to investigate age-dependent molecular responses to ischemic injury. The SAMR1/SAMP8 senescence-accelerated mouse model has been extensively characterized as a valuable experimental model to investigate age-associated cardiovascular dysfunction. Previous studies using this model have demonstrated early and progressive impairments in endothelial function [21], nitric oxide bioavailability [22], oxidative stress balance and inflammatory signaling [23], recapitulating key features of cardiovascular aging observed in humans. Importantly, these alterations develop in a reproducible and time-efficient manner, allowing mechanistic studies of aging-related cardiovascular pathophysiology processes under controlled experimental conditions and with reduced interindividual variability.

Nevertheless, it should be acknowledged that no single experimental model fully captures the complexity of human cardiac aging. Naturally aged animals, senescence-accelerated models, and disease-specific models each present inherent strengths and limitations. In this context, the SAMR1/SAMP8 model should be viewed as complementary to naturally aged models, providing a practical framework to dissect age- and sex-dependent molecular mechanisms, such as miRNA dysregulation, while minimizing confounding factors commonly present in clinical populations.

In line with our findings, age-dependent alterations in cardiac miRNA signaling have also been reported in naturally aged C57BL/6 mice and in human hearts. Studies comparing young (16 weeks) and aged (≈ 72 weeks) C57BL/6 mice have demonstrated that cardiac aging is associated with marked changes in myocardial miRNA content, including sex-dependent differences, and with enhanced profibrotic and inflammatory signaling [35]. Notably, these age-related miRNA alterations were conserved across murine and human samples, supporting the existence of core aging-related regulatory mechanisms maintained between species [35].

Moreover, age-dependent disruption of miRNA-regulated adaptive responses to myocardial ischaemia has also been described in naturally aged C57BL/6 mice. In this context, aging was associated with larger infarcts, worse contractile dysfunction, and impaired miRNA control of key contractile pathways after myocardial infarction [36]. Although focused on miR-146a-5p, these findings support the general concept that aging compromises miRNA-mediated compensatory programs following ischaemic injury. Together, these data indicate that the miRNA patterns identified in SAMP8 mice reflect general features of cardiac aging rather than model-specific phenomena, thereby strengthening the translational relevance of our findings.

The deregulation of miRNAs following a cardiovascular event has shown potential in the search of new cardiac biomarkers for over a decade [9, 37], and has been proposed as an accessible diagnostic tool. For example, Blanco-Domínguez et al. recently identified a novel miRNA that can be used to diagnose myocarditis [38], which is often misdiagnosed as myocardial infarction with non-obstructive coronary arteries. Using experimental mouse models of myocarditis and myocardial infarction, the authors identified mmu-miR-721 as a diagnose marker, that can be tested by liquid biopsy, avoiding the magnetic resonance imaging and invasive biopsy that are typically required.

We observed a significant increase in miRNA expression of the candidates miR-1-3p, miR-208a-3p, and miR-499-5p starting at 4 h post-ischemia and increasing further at 24 h. Therefore, the 4-hour time point may represent an optimal window to capture early circulating miRNA responses to AMI in this mice model. This time point also offers a practical experimental advantage, as mouse survival after LAD ligation is higher at early stages (approximately 90% at 4 h compared with ~ 80% at 24 h). Also, the 4-hour time point capture hyper-acute molecular responses to ischemic injury, prior to extensive inflammatory infiltration and structural remodeling, although additional time points will be required to fully define the temporal dynamics of cardiac miRNA regulation. Our results confirm those previously obtained in other studies. For example, Cheng et al. measured the levels of miR-1, miR-133a, miR-208 and miR-499 in the plasma of C57BL/6 male mice 6 h after inducing AMI, finding that all four miRNAs were upregulated [39]. These miRNAs have also been studied in patients with AMI, confirming the same trend as in mice for the overexpression of miR-1 and miR-133a [8], as well as for the overexpression of miR-208b and miR-499 [7]. All of these studies involved serum/plasma sampling within a limited time window of less than 12 h after the event. Evidence from existing studies indicates that miR-208b and miR-499 are highly expressed in heart tissue, while miR-1 and miR-133a are abundantly enriched in both heart and skeletal muscle [6, 8]. This knowledge correlates with our results from heart samples, in which all groups showed abundant expression of miR-1, miR-133, miR-208 and miR-499. Notably, heart miRNA expression decreases drastically in all AMI groups after the ischemic event. This is consistent with the findings of a similar study, which suggests that these miRNAs are released from heart after AMI and are transferred to different organs [39]. Our results demonstrate that non-ischemic aged heart have decreased expression of miR-499-5p in female mice and miR-133a-3p, miR-208a-3p, and miR-499-5p in male mice (Fig. 4). In contrast, other studies have found that the expression of miR-1, miR-133a, and miR-34 increases in the heart of older mice, with miR-1 showing an even greater increase in tissue expression in old mice after myocardial infarction [40].

Despite the largely comparable early post-infarction miRNA expression profiles observed between SAMR1 and SAMP8 mice, this does not preclude a meaningful influence of aging. At very early time points following myocardial infarction, conserved molecular stress-response programs are likely activated irrespective of age [41]. However, aging may critically modulate the cellular, vascular, and inflammatory context in which these miRNA changes occur [42], thereby influencing their downstream functional consequences. Thus, the preserved early miRNA response observed in SAMP8 mice suggests that aging does not abolish acute molecular activation, but may affect the efficacy, resolution, and long-term impact of these responses during subsequent cardiac remodeling.

Although sex-related differences in the pathobiology of AMI are well documented, they are not yet fully understood. Data show differences in the pathophysiology, presentation, diagnosis, treatment, and outcomes of AMI between women and men. These discrepancies are attributed to both biology and healthcare bias [43, 44]. Furthermore, sex differences in the expression of biomarkers have been observed in patients with myocardial infarction. In this context, Eggers et al. demonstrated that men exhibit higher levels of biomarkers indicative of cardiomyocyte necrosis and atherosclerosis promotion, whereas women exhibit greater concentrations of circulating proteins associated with the renin-angiotensin-aldosterone axis, inflammation, and adipokines [45]. Another study showed that the proteomic profile revealed sex-specific pathways, including inflammation, adiposity, fibrosis, and platelet homeostasis [46]. Moreover, the use of sex-specific threshold values in traditional diagnosis tests, such as cardiac troponins, remains limited [47]. Therefore, searching for new sex-specific miRNA biomarkers could be relevant for clinical use.

In support of this concept, sex-dependent regulation of miR-133a has also been described in other vascular beds. Cerebrovascular miRNA profiling in male and female 3xTg-AD mice identified miR-133a as a female-associated miRNA during early disease transitions [48], indicating that miR-133a expression can be sexually dimorphic in vivo. Although this study focused on brain vessels rather than myocardium, it supports the broader notion that sex-specific miRNA regulation represents a biologically relevant mechanism during aging and disease-associated vascular remodeling. Importantly, this sexually dimorphic regulation is supported by well-established functional roles of miR-133a in vascular homeostasis. miR-133a is a well-established regulator of vascular homeostasis, inhibiting caveolin-1 (CAV1) and thereby modulating endothelial nitric oxide synthase (eNOS) activity [49]. Its early downregulation may therefore contribute to impaired NO-mediated blood flow, a mechanism relevant to cardiovascular dysfunction. Importantly, estrogens have been shown to upregulate miR-133a, attenuating vascular remodeling by targeting transcriptional regulators [50].

We also observed sex-related differences in the basal expression of selected miRNAs in cardiac tissue, suggesting that the myocardial miRNA landscape is already sexually dimorphic before ischemic injury. These baseline differences may condition the post-infarction molecular response and could partly explain the sexually dimorphic regulation of circulating miR-133a-3p observed after myocardial infarction.

Moreover, the higher prevalence of some important risk factors such as diabetes mellitus, hyperlipidaemia, and hypertension in women compared to men is associated with an increased relative risk of AMI [43]. In this sense, our SAMR1/SAMP8 mouse model of AMI is useful for providing insight into the influence of sex and age in early transcriptional changes after ischemic injury. However, further studies using diet-induced protocols could evaluate other important risk factors, such as obesity and insulin resistance.

Some studies have investigated sex-specific miRNA in the heart. These are commonly attributed to the modulation of estrogen-driven transcriptional activity through specific receptors, as well as to the expression of sex chromosome-linked miRNAs that escape X-inactivation. The role of these miRNAs in different clinical outcomes after cardiac injury between women and men has been discussed, as has their potential use as sex-specific biomarkers [51]. In that sense, miR-21 and miR-106a/b have been identified as sex-specific miRNAs regulated via ERβ in a murine model of pressure overload-induced cardiac fibrosis [52]. In the context of AMI, Tsuji et al. [53] identified distinct miRNA expression patterns in male and female hearts under normal and ischemic conditions. In this study, miRNA profiling was analyzed using human post-mortem cardiac tissue and in mouse hearts one month after ischemic injury. miRNA showing sex dimorphism differed between normal and ischemic tissues, suggesting that sex differences in miRNA profiles change following myocardial injury. Specifically, thirteen miRNAs were identified as sexually biased in myocardial infarction in mice, including miR-505-5p, miR-744-5p, miR-210-3p, miR-30e-5p, miR-30b-5p, miR-29b-3p, miR-19b-3p, miR-193a-5p, miR-23a-5p, miR-142a-5p, miR-664-5p, miR-133a-5p, miR-214-3p. However, microarray data from human tissue revealed only three sex-associated miRNAs (miR-3615, miR-4423-5p, miR-4709-3p). To the best of our knowledge, no studies have reported sex-based differences in the expression of these miRNAs following myocardial infarction.

The enrichment of biological processes related to apoptosis, inflammation, and cell proliferation supports a functional involvement of the analyzed miRNAs in the early response to ischemic cardiac injury. These pathways are known to play a central role in cardiomyocyte survival, inflammatory signaling, and compensatory mechanisms following AMI. Consistent with our findings, previous studies have shown that miR-1 overexpression exacerbates cardiac ischemia/reperfusion injury, whereas its knockdown confers cardioprotection [54]. Similarly, miR-499-5p has been reported to protect cardiomyocytes against apoptosis following AMI [55], and upregulation of miR-208 has been associated with reduced myocardial damage, apoptosis, and inflammatory cytokine expression in experimental models of AMI [56]. Furthermore, the enrichment of pathways related to cardiac development, conduction, hypertrophy, and fibroblast proliferation suggests a potential role for these miRNAs in post-infarction, remodeling processes tightly linked to cardiovascular aging. Several of these pathways are known contributors to age-associated vascular and myocardial remodeling. In this context, reduced expression of miR-133a-3p, a well-established anti-hypertrophic and anti-fibrotic miRNA, has been associated with derepression of profibrotic signaling cascades, including TGF-β–dependent mechanisms [57, 58], thereby promoting fibroblast activation and extracellular matrix accumulation, hallmark features of cardiac aging. Likewise, miR-208a-3p and miR-499-5p, two cardiac-specific myomiRs encoded within myosin genes [59]), play central roles in the regulation of myosin isoform expression, stress adaptation, and the regulation of the heart conduction system [60]. Their age-associated downregulation may compromise metabolic efficiency, contractile coordination, and electrical stability, ultimately increasing susceptibility to functional decline and arrhythmogenic remodeling in the aging myocardium. Collectively, these observations support the notion that the miRNA alterations identified in this study reflect underlying mechanisms of cardiac aging and may modulate the myocardial response to ischemic injury through effects on inflammation, fibrosis, and structural remodeling.

This study has some limitations that should be acknowledged. First, due to the acute nature of the LAD occlusion model, infarct size could not be reliably quantified using non-invasive imaging techniques such as echocardiography or cardiac magnetic resonance. Although histological assessment would represent an alternative approach, this was not feasible because of sample size limitations and the prioritization of tissue for molecular and biochemical analyses aligned with the main objectives of the study. Nevertheless, myocardial infarction was consistently confirmed by characteristic electrocardiographic changes in all animals. Future studies using chronic models and larger cohorts will be necessary to allow accurate infarct size determination and to further validate the present findings. While functional and pathological analyses are required to establish a direct link between early miRNA changes (such as miR-1 upregulation) and myocardial infarction severity, the present study was specifically designed to focus on acute, age- and sex-dependent molecular responses during the early phase following ischemic injury.

In conclusion, our data demonstrate that AMI in SAM animals is successfully achieved with ligation of the LAD coronary artery, allowing us to evaluate the earliest transcriptional responses in the first hours after ischemic injury, and to elucidate the role of aging and sex differences in AMI pathophysiology. Further studies using this model to investigate changes in the miRNA profile and its role in transcriptomic changes after heart injury could provide insight into the sex differences observed in myocardial infarction in men and women of different ages.

## Supplementary Information

Below is the link to the supplementary material.

Supplementary Material 1

## Data Availability

No datasets were generated or analysed during the current study.
